# Cdk5 Inhibitory Peptide (CIP) Inhibits Cdk5/p25 Activity Induced by High Glucose in Pancreatic Beta Cells and Recovers Insulin Secretion from p25 Damage

**DOI:** 10.1371/journal.pone.0063332

**Published:** 2013-09-05

**Authors:** Ya-Li Zheng, Congyu Li, Ya-Fang Hu, Li Cao, Hui Wang, Bo Li, Xiao-Hua Lu, Li Bao, Hong-Yan Luo, Varsha Shukla, Niranjana D. Amin, Harish C. Pant

**Affiliations:** 1 Department of Nephrology, Ningxia People's Hospital, Yinchuan, Ningxia Province, China; 2 Pharmerit North America, Bethesda, Maryland, United States of America; 3 Laboratory of Neurochemistry, National Institute of Neurological Disorders and Stroke(NINDS), Bethesda, Maryland, United States of America; 4 Department of Neurology, Nanfang Hospital, Southern Medical University, Guangzhou, China; University of Pittsburgh, United States of America

## Abstract

Cdk5/p25 hyperactivity has been demonstrated to lead to neuron apoptosis and degenerations. Chronic exposure to high glucose (HG) results in hyperactivity of Cdk5 and reduced insulin secretion. Here, we set out to determine whether abnormal upregulation of Cdk5/p25 activity may be induced in a pancreatic beta cell line, Min6 cells. We first confirmed that p25 were induced in overexpressed p35 cells treated with HG and increased time course dependence. Next, we showed that no p25 was detected under short time HG stimulation (4–12 hrs), however was detectable in the long exposure in HG cells (24 hrs and 48 hrs). Cdk5 activity in the above cells was much higher than low glucose treated cells and resulted in more than 50% inhibition of insulin secretion. We confirmed these results by overexpression of p25 in Min6 cells. As in cortical neurons, CIP, a small peptide, inhibited Cdk5/p25 activity and restored insulin secretion. The same results were detected in co-infection of dominant negative Cdk5 (DNCdk5) with p25. CIP also reduced beta cells apoptosis induced by Cdk5/p25. These studies indicate that Cdk5/p25 hyperactivation deregulates insulin secretion and induces cell death in pancreatic beta cells and suggests that CIP may serve as a therapeutic agent for type 2 diabetes.

## Introduction

Cdk5 is a cyclin-dependent protein kinase which needs to associate with a regulatory partner, p35 or p39, for activation and maintaining numerous physiological functions in the nervous system including neurite outgrowth and axon patterning [1], [2]. Aberrant hyperactivation of Cdk5 induced by p25, a truncated fragment of p35, plays an important role in neurodegenerative diseases such as Alzheimer's disease (AD). Cdk5 hyperactivity leads to neuron apoptosis and degeneration [3], [4], [5], [6], [7]. Recent studies have documented the existence of both p35 and p39 in pancreatic beta cells and Cdk5 regulation of insulin secretion [8], [9], [10]. There are similarities between neurons and pancreatic beta cells, and between the neural degeneration of Alzheimer's disease and the deterioration of the beta cell functioning in type 2 diabetes [11], [12], [13]. Cdk5 activity also contributed to the pathogenesis of diabetes mellitus ([9], [14], [15], [16]. Glucotoxicity, which deteriorates functions of insulin on peripheral tissues and the secretion of insulin by beta cells is a critical factor of the pathophysiology of diabetes mellitus. In our previous studies, we showed that high glucose stimulates the increasing expression of p35 in Min6 cells and overexpressed p35 in HG, revealing the expression of p25, which led to decreased insulin secretion [17]. We also found a stressed neuron-like apoptosis with detection of p25 in the Min6 cells after exposing overexpressed p35 cells to a high glucose concentration [17]. Although the existence of p25 was clearly observed in our study, the role of Cdk5/p25 in pathogenesis to diabetes remains unclear; and whether inhibition of Cdk5/p25 activity can protect beta cells from pathology as it does with neurons.

To study this possibility, we confirmed the HG induced p25 expression in overexpressed p35 in Min6 cells, and then detected p25 accumulation as a function of time and glucose dosage in chronic glucose stress; as in hyperglycemia. Moreover, we over express p25 and cause over activations of Cdk5 in Min6 cells to see whether Cdk5/p25 activity results in inhibition of insulin secretion and cell apoptosis. Previously, we have shown that CIP, a smaller truncated peptide derived from p35, inhibits Cdk5/p25 activity in neurons without affecting Cdk5/p35 activity [18], [19]. A similar process may occur in glucose stressed beta cells. We co-infected of CIP with p25 in Min6 cells in order to study whether CIP inhibits Cdk5/p25 activity, restores insulin secretion, and rescues cells from apoptosis. These studies indicate that over-activation of Cdk5 by p25 may induce disruption of beta cells and deregulate insulin secretion. Accordingly, we suggest that the Cdk5 inhibitor, CIP, may serve as a therapeutic agent for type 2 diabetes.

## Materials and Methods

### Antibodies and reagents

Cdk5 (C-8), p35 (C-19) polyclonal antibodies (1∶1000), and anti-Myc (1∶500) polyclonal antibody were obtained from (Santa Cruz). Cleaved Caspase-3 (Asp^175^) antibody, Anti-tubulin antibody (Sigma, 1∶2000), Anti-β actin antibody (Sigma, 1∶1000) were purchased from cell signaling; Rat/Mouse insulin ELISA kit was obtained from Millipore.

### Generation of recombinant adenoviruses

The AdEasy system was kindly provided by Dr. Bert Vogelstein, Howard Hughes Medical Institute and Molecular Genetics Laboratory, Johns Hopkins Oncology Center, Baltimore, MD 21231, USA. A serial of Cdk5 related genes were generated by PCR with the following primer with additional restriction enzyme sequences underlined. 1. P35, forward primer, tttgcggccgccAtgggcacggtgctgtccct, reverse primer, tttgatatcttaccgatccaggcctagga; 2. p25 forward primer, tttgcggccgccAtggcccagcccccaccggccca, reverse primer is the same as p35. 3. Cdk5, forward primer, tttgcggccgccAtggagaaatacgagaaactgga, reverse primer, tttgatatcttagggcggacagaagtcgg. 4. CIP forward primer, tttgcggccgccAtgtgcctgggtgagtttctctg, reverse primer, tttgatatcttatgggtcggcatttatct. 5. Cdk5 dominant negative. Generation of recombinant adenoviruses was carried out according to the protocol (He et al). Subsequently, all PCR fragment were cloned into a shuttle vector, pAdTrack-CMV. The resultant plasmids were linearized and subsequently co-transformed into E. coli. BJ5183 cells with an adenoviral backbone plasmid pAdEasy-1(Strategy Com.). Recombinants were linearized and transfected into 293-1 cells (Strategy Com.) to generate the recombinant adenoviruses. High titer of virus were purified by CsCl banding and mixed with 2× storage buffer (10 mM Tris, pH 8.0, 100 mM NaCl, 0.1% BSA, and 50% glycerol, filter sterilized). Viruses were maintained as stocks at −20°C or −70°C. The titers of virus were checked by GFP when infected with related cells.

### Cell culture and transfecton

Mouse pancreatic beta cells Min6 [17],[20] and HEK293 cells were cultured in DMEM containing 25 mM glucose, 1 mM sodium pyruvate, 10% fetal bovine serum plus 100 µM penicillin G and 100 mg/ml streptomycin. Cells were infected independently or co-infected with empty vector (EV), the constructs of CIP (residues 154 to 279 of p35), p25 (residues 98 to 307 of p35), p35, wild-type Cdk5 and dominant negative Cdk5. Adenovirus vector packaging system was used as described earlier. After infection for 48 hrs, cells were starved overnight and treated with different concentrations of glucose (3 mM, 25 mM, and 50 mM) or aged Aβ_1–42_ (Sigma) incubated at 37°C for seven days before use at 10 µM for 6 hrs. The cells were fixed for immunohistochemistry analyses or lysed with lysis buffer for immunoprecipitation and Western blot analyses.

### Insulin Secretion Test

Beta cells were infected by expression adenovirus for 6 hours and the cell culture medium was replaced. After 48 hours transfection, cells were washed twice with basal Krebs-Ringer's solution bicarbonate Hepes (KRBH) buffer (3 mM glucose, 124 mM NaCl, 5.6 mM KCl, 2.5 mM CaCl2, 20 mM Hepes at pH 7.4, 0.5%BSA). The cells were then incubated with basal KRBH for 2 hrs. After discarding the medium, cells were incubated either with basal KRBH or KRBH containing high glucose (25 mM) for another 2 h. The supernants were collected to assay for secreted insulin. Cells were also harvested to assay for insulin content and for Western blots.

### Western blot analysis

Cells were harvested by scraping cells and lysed in ice-cold lysis buffer (20 mM Tris, pH 7.5, 150 mM NaCl, 1 mM EDTA, 1 mM EGTA, 1% Triton X-100, 1 mM β-glycerol phosphate, and 1 mM NaF, supplemented with a mixture of protease inhibitor cocktail and 1 mM PMSF), and incubated for 30 minutes on ice. After centrifugation for 20 minutes at 13,000× g at 4°C, the protein concentrations of the supernatants were determined using the BCA protein assay (Pierce, Rockford, Illinois). An equal amount of total protein (20 µg of protein/lane) was resolved on a 4–20%, a 15% or 8% SDS-polyacrylamide gel and blotted onto a PVDF membrane. The membrane was incubated in blocking buffer containing 20 mM Tris-HCl (pH 7.4), 150 mM NaCl, and 0.1% (v/v) Tween 20 (TTBS) plus 5% dry milk (w/v) for 1 hr at room temperature, and incubated with primary antibodies overnight at 4°C. The membranes were then washed four times in TTBS, followed by incubation in goat anti-mouse or goat anti-rabbit IgG (H+L)-HRP conjugated secondary antibodies (Amersham Biosciences, 1: 2500) for 2 hrs at room temperature. Western blots were analyzed using the Enhanced Chemiluminescence (ECL) kit (Pierce) following the manufacturer's instructions.

### Cdk5 kinase assays *in vitro*

Kinase assays were performed as previously described [21]. Briefly, Min6 cells were infected with p25, p35, Cdk5 wild-type, dominant negative-Cdk5 and CIP using the Adenovirus gene delivery system as described earlier. Cdk5 was immunoprecipitated from supernatants of lysed cells using the polyclonal C-8 antibody overnight at 4°C and immunoglobulin was isolated using Protein A-sepharose beads for 2 hours at 4°C. Immunoprecipitates were washed three times with lysis buffer and then once with 1× kinase assay buffer containing 20 mM Tris-Cl pH 7.4, 1 mM EDTA, 1 mM EGTA, 10 mM MgCl_2_, 10 µM sodium fluoride and 1 µM sodium orthovanadate. Kinase assays were performed in the same buffer containing 1 mM DTT, 0.1 mM ATP and 0.185 MBq [γ-^32^P] ATP with 20 µg of histone H1 as the substrate. Phosphorylation was performed in a final volume of 50 µl, incubated at 30°C for 60 minutes and stopped by addition of 10% SDS sample buffer and heated at 95°C for 5 minutes. Samples were separated by SDS-PAGE, gels were stained with Coomassie, destained, dried and exposed to autoradiography.

### Immunocytochemistry

Min6 cells were cultured and infected on glass coverslips. They were washed twice in PBS, fixed for 30 minutes at room temperature in 4% paraformaldehyde in PBS, and permeabilized with a buffer (25 mM Tris, pH7.4, 150 mM NaCl, and 0.1% Triton X-100) for 15 min. The coverslips were incubated overnight at 4°C with primary antibodies. All antibodies were diluted in PBS with 1% Triton-X-100. After three washes in PBS, coverslips were incubated with fluorescein goat anti-mouse IgG, or Texas Red goat anti-rabbit IgG for 1 hr at room temperature, followed by three washes with PBS. Cell nuclei were counterstained with Hoescht 33342 (Sigma) and then cells were mounted in aqueous medium (Biomeda). Fluorescent images were obtained with a Zeiss LSM-510 laser-scanning confocal microscope and images were managed with Adobe Photoshop.

## Results

### Overexpression of p35 in HG stressed Min6 cells induced p25 expression and increased time course dependence

**Figure 1:**
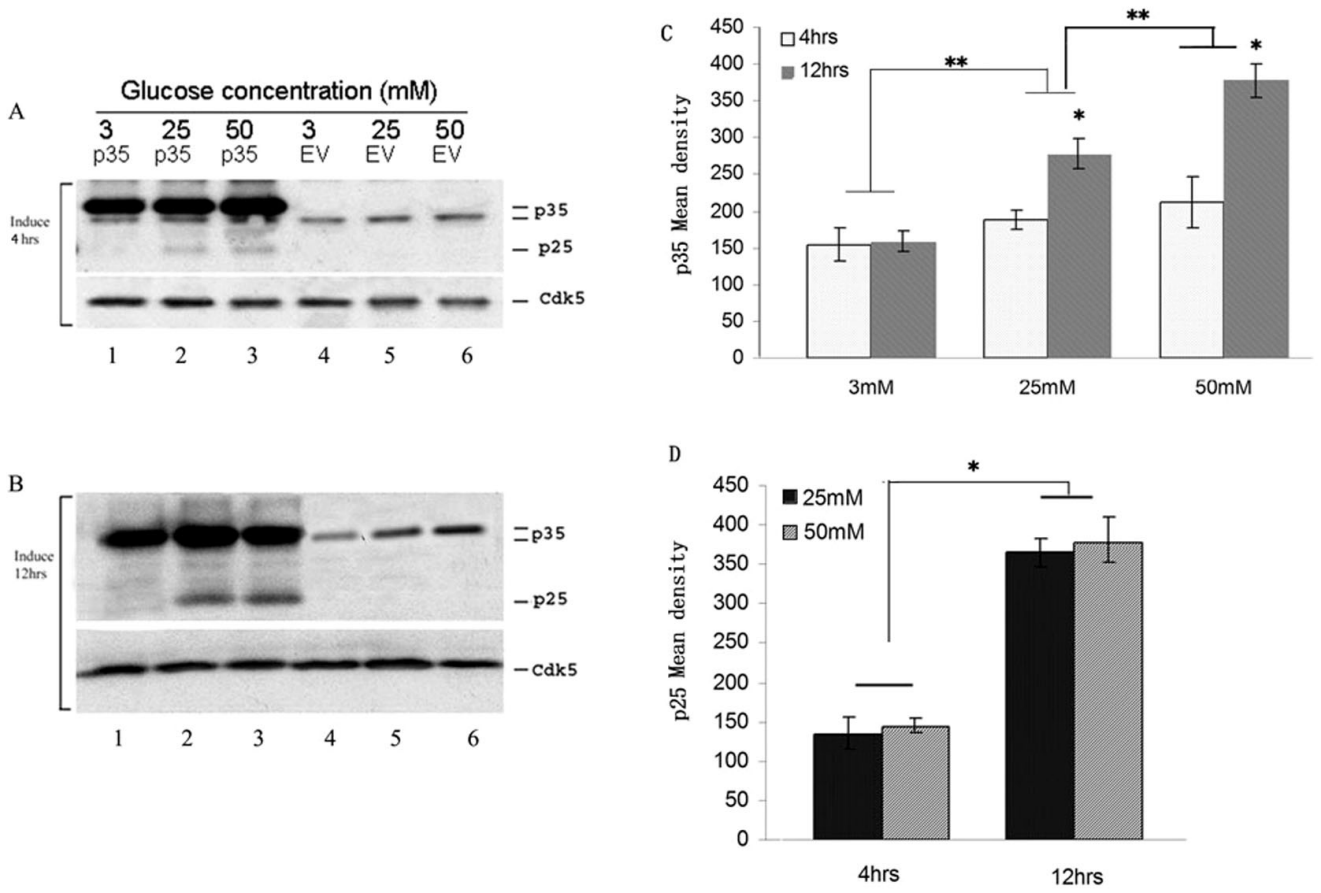
A time course and concentration course dependence of produced p25 expression in overexpression of p35 in HG stressed Min6 cells. A &B Overexpression of p35 produced p25 expression in Min6 cells stressed with HG (25 mM or 50 mM glucose) for 4 hrs (A, panel 1,lanes 2 and 3) and 12 hrs (B, panel 1, lanes 2 and 3). It exhibited a time dependence (Comparing A&B, penal 1, lanes 2 and 3). Cdk5 expressions were not affected by about stimulations (A&B, panel 2, lanes 1–6). C Quantification of Overexpression of p35. D Quantification of p25 expression produced by overexpressing of p35 plus stressed with HG in Min6 cells. (*shows time course comparison; ** shows dosage course comparison). Data represent mean ± SE of three experiments.

Our previous study has showed that overexpressing p35 in Min 6 cells treated with high glucose induced p25 expression. We first confirmed these results in Figure 1. p25 formation from endogenous p35 was not detected in low or high glucose exposure for 4 h or 12 h as seen in Fig. 1A (lane 4–6). In p35 overexpressed cells, however, treated with HG (25 mM or 50 mM glucose), p25 produced and exhibited a time course dependence (Fig. 1A, 1B lanes 1–3). Fig. 1C showed the endogenous amount of p35 expressions in Min6 cells stressed with HG, which increased time and dosage course dependence (p<0.01). Fig. 1D showed the amount of p25 produced from overexpressed p35 min6 cells stressed with HG (25 mM and 50 mM) at a different time (4 and 12 hrs). p25 productions were significantly increased in the 12 hrs (compared to 4 hrs, p<0.01). Cdk5 expressions were not affected by stimulations (Fig. 1A&B, lanes 1–6).

### Generation of p25 in Min6 cells under chronic high glucose stimulation

**Figure 2:**
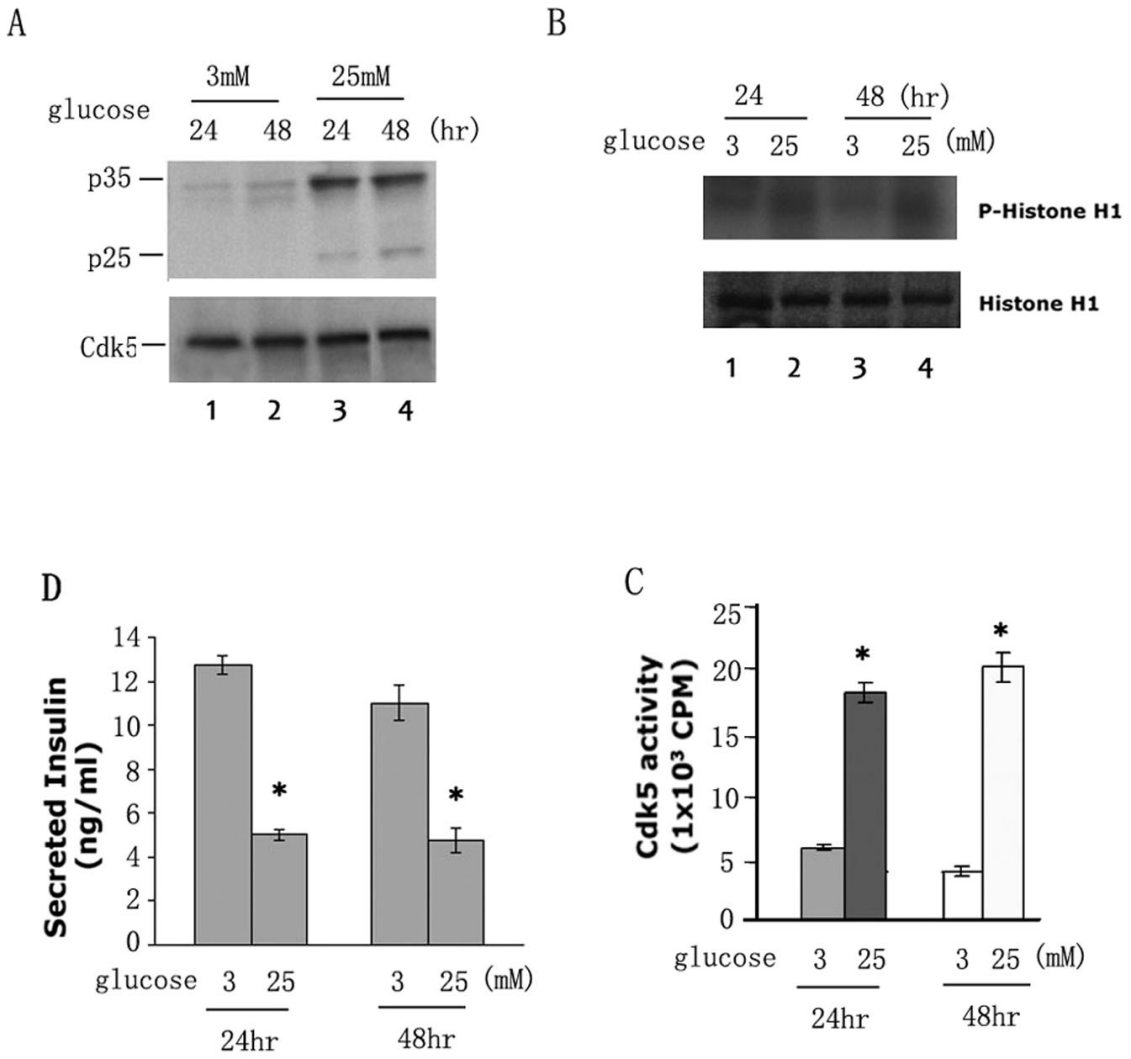
Chronic high glucose stimulation induces p25 expression in Min6 cells. A Endogenous p25 expression induced by high glucose (25 mM) (panel 1, lanes 3 and 4.) B Autoradiograph of phosphorylated histone H1 in the glucose induced Min6 cells (panel 1, lanes 2 and 4). The same cell lysates were used for the kinase assay using Cdk5 immunoprecipitates. C Quantification of Cdk5 activities in the glucose induced Min6 cells. D Insulin secretion assay. The insulin secretion was significantly inhibited in the high chronic glucose treated cells compared to the low glucose treated cells (* P<0.01). Data represent mean ± SE of three experiments.

We asked if p25 is produced from endogenous p35 in Min6 cells by chronic glucose exposure. Min6 cells were cultured and starved KRBH (glucose free) overnight and treated with 3 mM and 25 mM glucose respectively for 2 hrs or 14 hrs. Harvested cells were prepared for Western Blots. As suspected, p25 formation from endogenous p35 was not detected in low or high glucose exposure for 2 hrs or 12 hrs which were showed in Fig. 1A&B, lane 4–6. However, extending the exposure time to 24 hrs and 48 hrs, p25 was induced in cells exposed only to high chronic glucose (Fig. 2A, lanes 3–4). Under the conditions of this experiment, we can induce the deregulation of Cdk5 in a manner resembling that seen in stressed neurons; i.e., cleavage of p35 to p25. Cdk5 activity after 24 hrs and 48 hrs exposure in the 25 mM glucose treated cells is greater than the activity in cells at 3 mM glucose (Fig. 2B, compare right lane and left lane, p<0.01) suggesting the presence of the more active Cdk5/p25. After 24 and 48 hrs treatment, the insulin secretion in 25 mM glucose treated cells decreased more than 50% in comparison to insulin secretion in 3 mM glucose treated cells (Fig. 2C, p<0.01).

### Over-expression of p25 in Min6 cells causes hyperactivity of Cdk5 and inhibits insulin secretion

**Figure 3:**
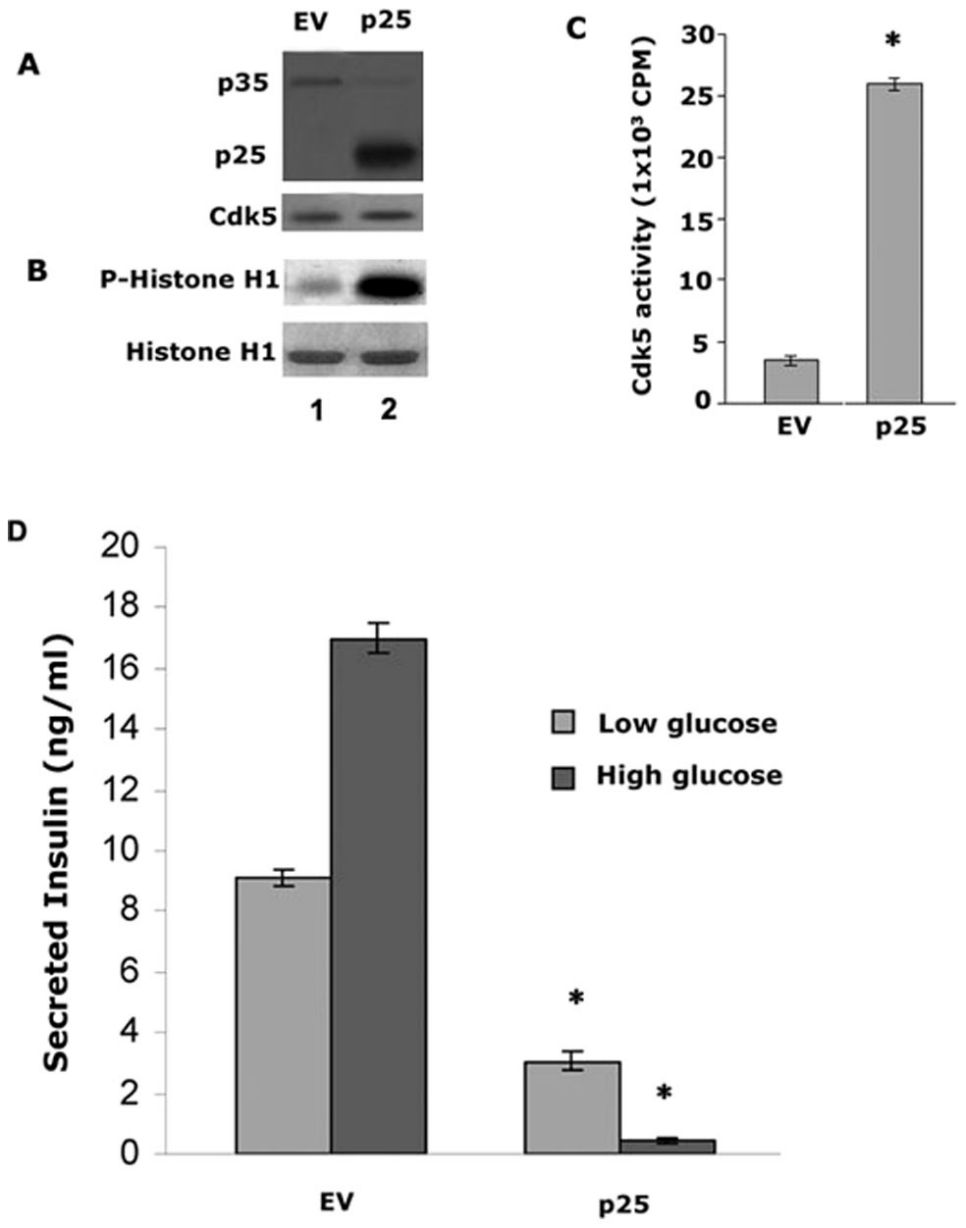
Min6 cells infected with p25, display high levels of Cdk5 activity and inhibits insulin secretion. A Expressions of infected p25 (panel 1, Lane 2) and endogenous p35 (panel 1, lanes 1 and 2) and Cdk5 (panel 2 lanes 1 and 2). B Autoradiograph of phosphorylated histone H1 in infected Min6 cells. The same cell lysates were used for the kinase assay using Cdk5 immunoprecipitates. C Quantification of Cdk5 activities in infected Min6 cells. Data represent mean ± SE of three experiments. D Insulin secretion was extremely inhibited when p25 were overexpressed in Min6 cells. Insulin released from p25 infected cells was dramatically inhibited under basal and stimulated conditions, more than 95% in the latter case, (* P<0.01). Data represent mean ± standard error of three experiments.

As discussed previously, the effect of Cdk5 activity on insulin secretion is controversial; it either inhibits or promotes insulin secretion depending on glucose concentration [10], [22]. To confirm that deregulation of Cdk5 by p25 deregulates insulin secretion, we studied the effect of Cdk5 hyperactivity induced by overexpression of p25 in Min6 cells. The Western blot in Fig. 3A showed the extent of overexpression of p25 as well as the level of endogenous Cdk5 expression. In Fig. 3B & C, we compared Cdk5 activity in immunoprecipitates from infected Min6 cells with EV or p25 and showed that Cdk5 activity in over expressing p25 cells exhibited a five-fold increase over controls (Figs. 3B & C, right lane). To determine the effect of Cdk5 activity on insulin secretion, two groups of infected Min6 cells (EV and p25) were cultured and starved with KRBH buffer for 2 hrs, then stimulated with low glucose (3 mM) and high glucose buffer (25 mM) for 2 hrs respectively. As seen in Fig. 3D, insulin secretion in control cells (EV) is 2-fold greater in high glucose than in low glucose. In low glucose, p25 infected cells exhibit a three-fold decline in insulin secretion compared to controls (p<0.01), whereas in high glucose, insulin secretion was dramatically reduced to 90% of controls in p25-infected cells (p<0.01). Insulin secretion seems to be inversely proportional to the level of Cdk5 activity in both low and high glucose respectively, in Min6 cells infected with p25.

### CIP specifically inhibits Cdk5/p25 activity but not Cdk5/p35 activity in Min6 cells

**Figure 4:**
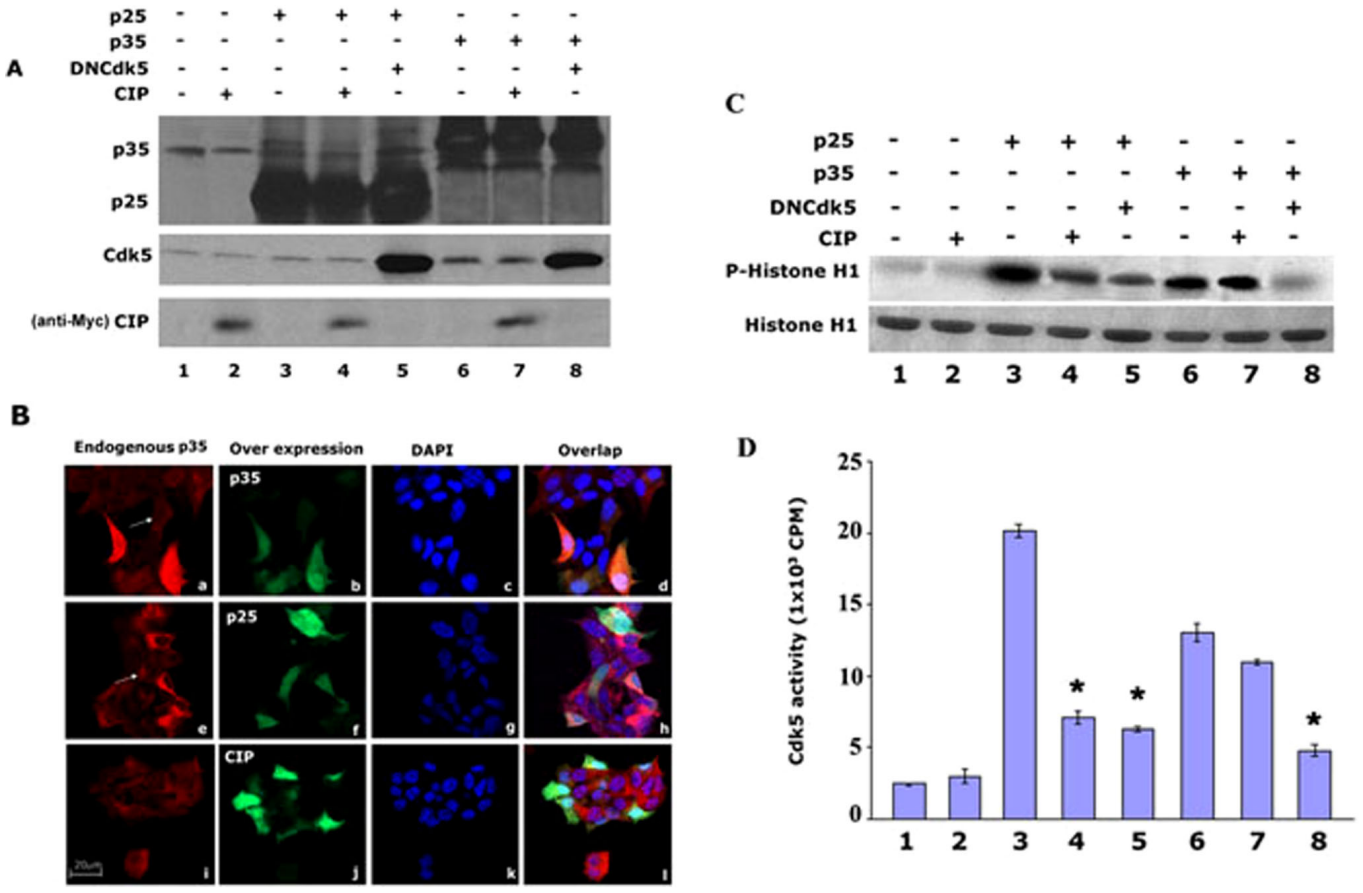
CIP specifically inhibits Cdk5/p25 activity in co- infected Min6 cells.

Our previous study showed that roscotitine, a Cdk5 inhibitor, inhibits Cdk5 activity and prevents Min6 cells from apoptosis, and restores insulin secretion ([17]. However, the inhibition of roscovitine is universal for all Cdks. CIP, a smaller truncated peptide derived from p35, specifically inhibits Cdk5/p25 activity as in cortical neurons [18], [19]. It did so without affecting endogenous Cdk5/p35 activity and other Cdks activities. To explore the possibility that CIP has a similar effect on glucose stressed Min6 cells, we co-infected cells with p25, p35 together with or without CIP, and dominant negative Cdk5 in combinations shown in the expression levels depicted in Fig. 4A. Overexpression of p25 showed at lanes 3–5 and overexpressed p35 showed at lanes 6–8 of upper panel; over expressed DNCdk5 showed at lanes 5 and 8 of middle panel; and CIP was showed at lanes 2, 4, and 7 of low panel. The cytosolic location of endogenous p35 was shown in Fig. 4B, (a, e, and i) while the location of exogenous p35 and p25 is seen in Fig. 4B, (b, f, and j). The distribution of Cdk5 activities in the above cells is shown in Fig. 4C&D. We see that activation of Cdk5 by endogenous p35 (lane 1) is unaffected by infected CIP (lane 2). In lane 3 we note that Cdk5 is hyperactivated by overexpressed p25 and was effectively inhibited by CIP (lane 4), which was confirmed by co-infected p25 plus DNCdk5 (lane 5). Over expression of p35 showed that increased Cdk5 activity (lane 6) can be inhibited by DNCdk5 (lane 8), but not CIP (lane 7).

### CIP recovered insulin secretion caused by over-expression of p25 in Min6 cells

**Figure 5:**
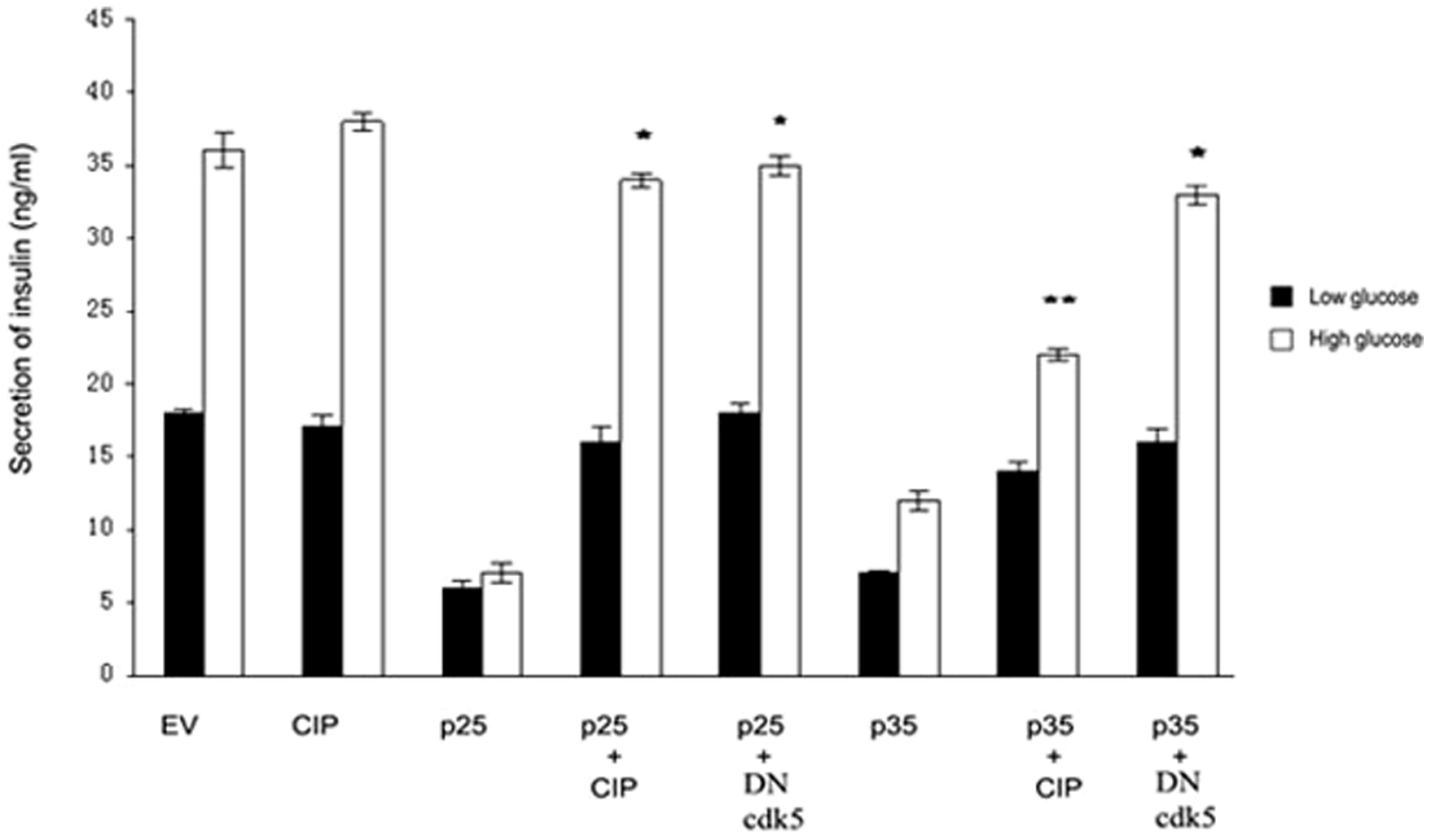
CIP restores insulin secretion after inhibition by elevated Cdk5 activity. Min 6 cells were transiently infected with EV, CIP, p25, p25/CIP, and p25/DNcdk5 constructs respectively. After 48 hours infection, cells were washed and starved for 2 hrs. Then, cells were stimulated by 3 mM and 25 mM glucose respectively for 2 hrs. Insulin secreted into the supernatant was measured using LINCO, ELISA Kit. CIP increased the insulin secretion after inhibition by p25 (compared p25/CIP to p25), (* P<0.01; ** P<0.05). Data represent mean ± SE of three experiments.

To determine whether CIP inhibition of hyperactive Cdk5/p25 effects insulin secretion, we compared insulin secretion in Min 6 cells transfected with different vectors similar to those used in Fig. 5. Transfected cells were stimulated by low (3 mM) and high glucose (25 mM) for 2 hrs. As shown in Fig. 5, insulin secretion in control cells increased four times under high glucose stimulation compared to low glucose stimulation (lane EV). CIP alone did not effect insulin secretion in these cells, presumably expressing elevated levels of Cdk5/p35 activity [9]. On the other hand, infected p25 dramatically inhibited insulin secretion in two glucose conditions, 3 and 5-fold respectively (lane p25), but p25 co-expressed with DNCdk5, restored insulin secretion to control cell levels (lane p25+DN). What is most striking is that co-infection of CIP with p25, resulted in a similar rescue of insulin secretion to control levels (lane p25+CIP). Interestingly, CIP also recovered insulin secretion in cells overexpressing p35 (lane p35+CIP), this further indicates that regulation p35 on insulin secretion may be produced p25. These results further confirm that CIP inhibits hyperactive Cdk5/p25 activity in Min6 calls as it does in stressed neurons and restores normal levels of insulin secretion in beta cells stressed by chronic glucose stimulation, as in hyperglycemia, a condition seen in T2DM.

### High glucose treatment induced apoptosis in Mim6 cells can be rescued by CIP

**Figure 6:**
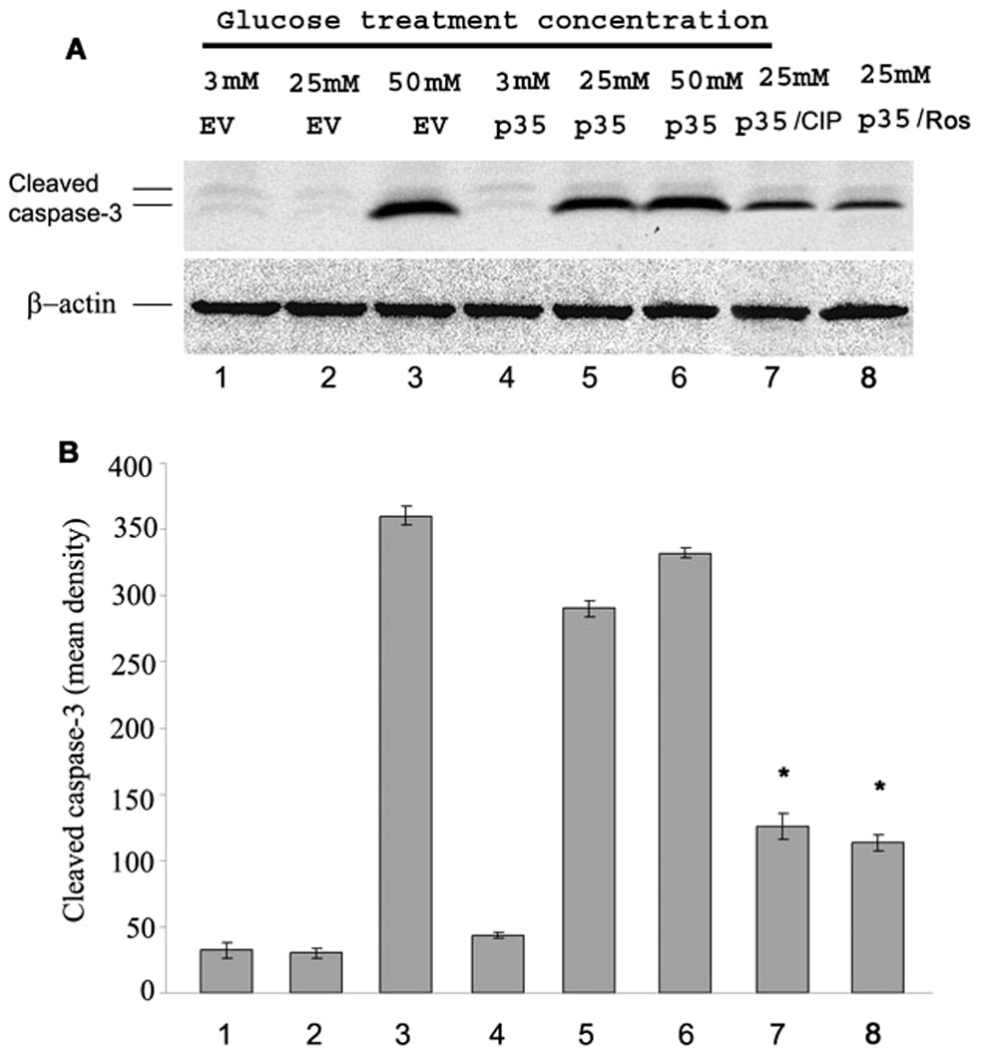
CIP can rescued high glucose treatment induced apoptosis in overexpressed p35 Min6 cells. A Cells infected with p35 were apoptotic at both 25 mM and 50 mM glucose concentrations (lanes 5 and 6). Cells with co-transfected CIP however, showed a reduced level of apoptosis (lane 7), and the same result can be see in the cells treated with roscovitine (lane 8). The control cells at HG (50 mM) showed a strong cleaved caspase 3 band which indicated that very high glucose is indeed cytotoxic. Interestingly, there is no apoptosis markers in the cells infected with p35 and treated with 3 mM glucose (lane 4). B The apoptosis data quantified from A, (* P<0.01). Data represent mean ± SE of three experiments.

Cortical neurons are induced to apoptosis in the presence of hyperactive Cdk5/p25. Such cells however can be rescued by co-infection with CIP [18], [19]. To observe whether high glucose treatment is cytotoxic and causes apoptosis, Min6 cells were infected with EV or p35 with or without CIP and treated with 3 mM, 25 mM, and 50 mM glucose respectively for 4 hrs. Cells were harvested, lysed and submitted for Western Blot analysis using anti- cleaved caspase-3 antibody as an apoptosis marker. In Fig. 6A, control cells at high glucose (50 mM) were apoptotic as indicated by a strong caspase 3 band; clearly very high glucose is indeed cytotoxic. Cells infected with p35 however, were apoptotic at both 25 mM and 50 mM glucose concentrations (lanes 5 and 6) suggesting that elevated levels of Cdk5/p35 (and/or Cdk5/p25) activity is significantly more cytotoxic. Cells with co-transfected CIP however, showed a reduced level of apoptosis (lane 7), as same as in the cells treated with roscovitine (lane 8). Interestingly, there is no evidence of apoptosis in the cells infected with p35 and treated with 3 mM glucose (figure 6A, lane 4).

## Discussion

Cdk5 plays an important role in regulation of insulin secretion by increasing p35 gene expression in high glucose [8], [9], [10], [13]. Transient elevations of extracellular glucose promote pancreatic beta cell function and survival [23], [24]. Chronic elevations of glucose however, has the opposite effect, impairing beta -cell function and survival [25], [26]. The deleterious effects of chronically elevated glucose are referred to the glucotoxicity. Recent studies have shown that increasing endogenous p35 expression inhibits insulin secretion by activating the Cdk5 activity in beta-cell lines [9], [13]. The mechanism remains unclear. Our previous study found that Cdk5 upregulated in p35 over expressed Min6 beta cells stimulated by high glucose inhibits insulin secretion [17]. In this study, we further explored the role of Cdk5/p25 in pathogenesis to insulin secretion in pancreatic beta cells (Min 6 cells) and whether the pathogenesis can be inhibited by Cdk5 inhibitory peptide, CIP as it happened in degenerative neuronal cells [19].

Chronic exposure of INS-1 cells to high glucose without the appearance of p25 results in elevated Cdk5/p35 activity and reduction in insulin expression [9], [22]. Our previous study of time course HG stimulation on Min6 also showed the same results [17]. However, we also found that upregulation of p25 and Cdk5 activity in Min6 cells over expressing p35 dramatically inhibits insulin secretion in high glucose [17]. These results suggest that HG may induce undetectable levels of p25 from upregulated endogenous p35, possibly an initial stage in glucose toxicity.

Failure to detect endogenous p25 expression in cells in low and high glucose at 4 hrs to 14 hrs [17] is probably due to (1) the low levels of endogenous p35 and (2) the short term exposure. In order to confirm the above hypotheses in this study, we first produced the expression of p25 in over expressed p35 in Min6 cells treated with HG (Figure 1). Then, we performed a time course of HG treatment study using Min6 cell and demonstrated that p25 will be induced from endogenous p35 after 24 hrs HG stimulation (Figure 2). Both of two conditions inhibit insulin secretion by hyper activity of Cdk5. Above results indicate that p25 is induced by chronic glucose toxicity stressed beta calls. We hypothesized that the over activated Cdk5 by p25 inhibits insulin secretion and may play an important role in T2DM.

P25 is cleaved from p35, which binds and hyperactivates Cdk5. As a consequence of p35 cleavage, Cdk5 forms a stable, long-lived, hyperactive Cdk5/p25 complex that induces cytoskeletal disruptions and cell death. A similar series of events is induced in beta cells in high, toxic glucose with a consequent inhibition of insulin synthesis [22] and secretion, leading to beta cell pathology and cell death, a degenerative process resembling that seen in type 2 diabetes (T2DM). That cleavage of p35 to p25 may occur in islet cells in high glucose was initially suggested by the observation that Cdk5 activity is translocated from a membrane bound fraction to the cytosol promoting insulin secretion [8]. This, of course runs counter to the hypothesis that a more active Cdk5 should inhibit insulin secretion as our expression data demonstrate.

Our data are consistent with Cdk5 deregulation via cleavage of p35 to p25, hyperactivation of Cdk5/p25. Our studies rely on Min6 cells in which p35 and p25 are overexpressed by transfection. Here, in both low and high glucose, several key events occur; (1) Cdk5/p35 and Cdk5/p25 activities are significantly activated; (2) p25 is upregulated in cells overexpressing p35 suggesting cleavage of the latter in low and high glucose after 2–12 hrs stimulation; (3) Insulin secretion is inhibited; (4) apoptosis occurs in p35-overexpressed cells in chronic glucose exposure (25–50 mM for 4 hrs, Fig. 6), or endogenously in 50 mM. The data suggest that increased Cdk5 activity in cells overexpressing p35 in low and high glucose reflects cleavage of p35 to p25 resulting in the combined activities of Cdk5/p35 and cdk5/p25. The former probably localized at the cell membrane, the latter soluble in the cytosol and significantly more active.

Cells under these conditions may be in a state of glucotoxicity [9]. Here, glucotoxicity was induced at high glucose for 48 hrs and cells were rescued by inhibiting Cdk5 activity [9]. Indeed, this is a strong possibility since cells overexpressing p35 (and elevated Cdk5 activity) in high glucose for 4 hrs undergo apoptosis as if exposed to chronic glucose stimulation. We suggest that here too, both Cdk5/p35 and Cdk5/p25 activities are responsible for apoptosis. The CIP (Cdk5 Inhibitory Peptide) inhibition data are consistent with this hypothesis.

The effect of the inhibitor CIP on cells overexpressing p35 and p25 as well as its effect on cells experiencing glucotoxicity support the model. In cortical neurons, CIP, a truncated fragment derived from p35 specifically inhibits Cdk5/p25 activity, tau hyperphosphorylation and cell death without affecting cdk5/p35 activity [18], [19], [27]. A similar effect is seen in Min6 cells overexpressing p35 and/or p25 with CIP inhibiting Cdk5/p25 activity, rescuing insulin secretion, (Fig. 5) and inhibiting apoptosis (Fig. 6), without affecting Cdk5/p35 activity. As we have demonstrated, overexpression of p35 does lead to p25 induction, which means that the presence of both active complexes would result only in CIP inhibition of Cdk5/p25 leaving an unaffected cdk5/p35 complex with residual activity (compare lane p35 with p35 + CIP, Fig. 5) and (lane 4 with lane 7, Fig. 6). In both cases, the effect of elevated Cdk5/p35 activity on inhibition of insulin secretion and apoptosis persists but at a reduced level.

The results of CIP inhibition of glucotoxicity are most compelling. Here the principle effect on Cdk5 hyperactivity, inhibition of insulin secretion and apoptosis can be attributed almost exclusively to upregulated p25 (p35 levels are much lower under these conditions) and the dramatic inhibition by CIP of the abnormal phenotypes is remarkably similar to its rescue of challenged cortical neurons. This raises the possibility that Cdk5 can serve as a therapeutic target for both neurodegenerative disorders such as AD and diabetes. In a series of recent studies it has been well established that AD and T2DM are linked metabolically and pathologically in a number of ways [11], . They share such abnormalities as impaired glucose metabolism, increased oxidative stress, amyloid deposition and insulin resistance. Our results go much further in establishing the similarity between neurons and insulin secreting beta cells; they show that deregulation of Cdk5 in Min 6 cells follows the pattern of Cdk5 deregulation in neurodegenerative disorders. CIP, a specific inhibitor of hyperactive Cdk5/p25 rescues both stressed neurons and insulin secreting beta cells, may serve as an effective therapeutic agent for both disorders.
